# Ancient Danish Apple Cultivars—A Comprehensive Metabolite and Sensory Profiling of Apple Juices

**DOI:** 10.3390/metabo9070139

**Published:** 2019-07-11

**Authors:** Nunzia Iaccarino, Camilla Varming, Mikael Agerlin Petersen, Nanna Viereck, Birk Schütz, Torben Bo Toldam-Andersen, Antonio Randazzo, Søren Balling Engelsen

**Affiliations:** 1 Department of Pharmacy, University of Naples Federico II, Via D. Montesano 49, 80131 Naples, Italy; 2 Novozymes, Biologiens Vej 2, 2800 Kongens Lyngby, Denmark; 3 Department of Food Science, University of Copenhagen, Rolighedsvej 26, 1958 Frederiksberg C, Denmark; 4 Bruker BioSpin, Silberstreifen 4, 76287 Rheinstetten, Germany; 5 Department of Plant and Environmental Sciences, University of Copenhagen, Hoejbakkegaard Alle 13, 2630 Taastrup, Denmark

**Keywords:** apple juice, ancient apple cultivars, Bruker-SGF Fruit Juice Screener, NMR, sensory analysis, HS-GC/MS

## Abstract

In recent decades, intensive selective breeding programs have allowed the development of disease-resistant and flavorsome apple cultivars while leading to a gradual decline of a large number of ancient varieties in many countries. However, the re-evaluation of such cultivars could lead to the production new apple-based products with health beneficial properties and/or unique flavor qualities. Herein, we report the comprehensive characterization of juices obtained from 86 old, mostly Danish, apple cultivars, by employing traditional analysis (ion chromatography, °Brix, headspace gas chromatography/mass spectrometry (GC–MS), and panel test evaluation) as well as an innovative nuclear magnetic resonance (NMR)-based screening method developed by Bruker for fruit juices, known as Spin Generated Fingerprint (SGF) Profiling™. Principal component analysis showed large differences in aroma components and sensory characteristics, including odd peculiar odors and flavors such as apricot and peach, and very different levels of phenolic compounds, acids and sugars among the analyzed juices. Moreover, we observed a tendency for late-season juices to be characterized by higher °Brix values, sugar content and they were perceived to be sweeter and more flavor intense than early-season juices. Our findings are useful for the production of specialty vintage-cultivar apple juices or mixed juices to obtain final products that are characterized both by healthy properties and peculiar sensory attributes.

## 1. Introduction

Apple (*Malus domestica*) juice consumption is widely spread worldwide, and it is supposed to grow in coming years [1], probably due to its specific pleasant taste and potential health benefits [2]. In particular, European populations seems to prefer apple juice over peach, pineapple and other fruit juices. This is in line with the juice market in Denmark, where the apple juice represents 29.7% of total juice consumption, being the third most consumed juice after orange juice and flavor mixes. Focusing on Danes’ habits, a very interesting trend has been observed in the past few years: people drink less juice but of better quality, thus the demand for premium products grows [3].

**Figure 1:**
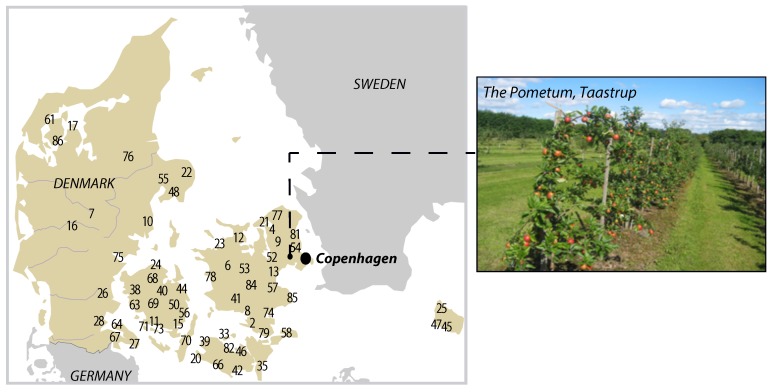
Geographical location of the University of Copenhagen ‘Pometum’ that hosts the collection of national and international fruit genotypes. The numbers on the map refer to the sample number in Table 1 and show the place of origin of the Danish cultivars.

In recent decades, apple cultivars such as Gala, Fuji, Braeburn, Honeycrisp, Pink Lady, and Empire have become more popular among consumers all over the world. These cultivars are often the result of intensive selective breeding programs developed in order to improve disease resistance and shelf life of the fruit together with its sensory attributes. This process has led to a gradual decline in the growing of a large number of ancient varieties. However, in the last few years, a strong tendency to re-evaluate these ancient apple cultivars has appeared, for example in Italy [4,5], the United Kingdom [6], Croatia [7], Poland [8] and Germany [9]. These studies are mainly conducted with the aim of developing new apple-based products with health beneficial properties and/or unique flavor qualities that can be attractive to an emerging segment of consumers. Denmark, and in particular, the University of Copenhagen, has dedicated huge efforts to the study of local apple cultivars. A vast apple orchard and gene bank collection called ‘The Pometum’, located in Taastrup (Denmark) (Figure 1), comprises about 750 apple varieties of which approximately 250 are of Danish origin. Until recently, many of these cultivars were only characterized with respect to phenotypic parameters that are publicly available on the Pometum website [10]. However, lately, also a genotype fingerprinting of 448 of these cultivars was performed by Larsen and co-authors to investigate their ancestral geographical origin [11]. In this frame, we decided to provide, for the first time, an extensive chemical, aroma, and sensory characterization of 86 apple cultivars harvested from the Pometum by employing a combination of analytical techniques such as nuclear magnetic resonance (NMR)-based juice screening, dynamic headspace gas-chromatography-mass spectrometry (GC–MS), ion-chromatography and sensory evaluation. With the help of multivariate data analysis, we searched for correlations and differences among the data of different nature as well as clusters of cultivars having similar chemical and/or unique sensory properties, with the aim to identify cultivars for the production of ‘vintage juices’ for niche markets.

In Denmark, apples are normally harvested between late August and mid-October depending on cultivar and weather conditions. Late-ripening apple cultivars may have better conditions for development of flavors and other substances; however, whether there is an association between the time of ripening of a cultivar in the season and the chemical and sensory qualities of the juice, has, to the best of our knowledge, not been reported in the literature. Thus, an additional aim was to investigate how juices of early and late cultivars differ with respect to their chemical and sensory parameters.

## 2. Materials and Methods 

### 2.1. Fruit Material and Juice Preparation

Apples (*Malus domestica*) of 86 varieties were harvested in 2010 at maturity at the experimental orchard and gene bank ‘The Pometum’ belonging to Copenhagen University. Each apple cultivar was harvested from two apple trees at optimal picking time. Since most of the apple cultivars are not commercially grown, there are no standard values for optimal picking time. However, experienced staff with knowledge of the apple cultivars assessed the optimal picking time of the apples upon kernel color, taste, peel color, and ease of picking. A random sample consisting of 10–20 healthy fruits, from all over the trees, was picked. The apples were stored at 4 °C for 0 to 10 weeks, depending on the post-harvest ripening requirement of the individual cultivars based on continuous evaluation of texture and taste of the apples. The fruits were then washed, cut with a Braun Food processor and pressed in a stainless steel 20 L hydro-press (Speidel, Germany). Juices for chemical analyses were bottled in 25 mL white polyethylene bottles (ApodanNordic PharmaPackaging A/S, Copenhagen, Denmark) and immediately frozen (–18 °C). Juices for sensory evaluation were bottled in 500 mL white polyethylene bottles (ApodanNordic PharmaPackaging A/S, Copenhagen, Denmark), and to ensure microbiological safety, the bottled samples were pasteurized in a micro-wave oven (2.5 min at 900 watt) to a temperature of approximately 80 °C. Samples were rapidly cooled in an ice/water bath and stored at 2 °C. The sensory evaluations were performed after approximately four months of storage.

In order to evaluate differences between juices of early and late cultivars, the harvest day of each apple cultivar was registered. The first apple cultivars were harvested on 23 August, and the last cultivars were harvested 52 days later. Juices were divided into two groups according to harvest day i.e., harvest day 1–30 (23 August–21 September) was assigned to class ‘early’, while harvest day 31–52 (22 September–14 October) was assigned to class ‘late’. The former group contained 39 cultivars, while the latter contained 47 cultivars. All the details about the cultivars’ characteristics are reported in Table 1.

### 2.2. Sugar and Acid Measurements: Brix and Ion Chromatography

Sucrose, glucose, fructose, malic, citric and succinic acid were measured using ion chromatography (IC). After thawing, 1.0 mL juice was filtered through a 25 mm Q-Max syringe filter with a 0.45 µm (pore size) cellulose acetate membrane (Frisenette Aps, Knebel, Denmark) and diluted 50 times with distilled water. The sample was injected on a Compact IC Pro model 881 combined with a Basic IC plus 883 unit equipped with a split port for simultaneous analysis of sugars and acids. For carbohydrate separation, a Metrozep C4-250/4.0 cation column was used, and for organic acids, a Metrozep Organic Acids –250/7.8 column was used. Detectors were an IC amperometric detector and a conductivity detector for carbohydrates and organic acids, respectively. The system was combined with a 919 IC auto sampler plus. All units were from Metrohm AG, Switzerland and controlled with ‘MagIC Net’ professional software. Calibration curves were made for glucose, fructose, sucrose and malic, citric and succinic acid using pure analytical grade solutions. °Brix was measured in one drop of juice using a QUICK°BRIX60 (Mettler Toledo, Glostrup, Denmark) portable °Brix meter. Analyses were made as single determinations, however, a measure of the IC accuracy was obtained by analyzing seven replicates of a standards’ mixture (see ‘Analytical Platforms Reliability’ in Supplementary Material).

### 2.3. Analysis of Aroma Compounds by Dynamic Headspace Gas Chromatography/Mass Spectrometry (GC–MS)

Samples were thawed over night at 5 °C. 15 mL of apple juice was pipetted into a 100 mL glass flask, and 1.00 mL of internal standard (50 µL L^−1^ 4-methyl-1-pentanol (97%), Aldrich, Steinheim, Germany) was added. The use of internal standard allowed us to control sampling and analysis was unproblematic, i.e., the internal standard peak areas were in the same range in all chromatograms. The glass flask containing the sample was equipped with a purge head and transferred to a water bath, where the sample temperature was equilibrated at 30 °C for 10 min. Under magnetic stirring (200 rpm), the sample was then purged with nitrogen (100 mL min^−1^) for 20 min, while the volatiles were collected in traps containing 250 mg of Tenax TA (mesh size 60/80, Buchem BV, Apeldoorn, The Netherlands). Dynamic headspace collections for gas chromatography/mass spectrometry (GC–MS) were carried out as single determinations. For details about the CG-MS accuracy, please see ‘Analytical Platforms Reliability’ in Supplementary Material.

The collected volatiles were thermally desorbed using an Automated Thermal Desorber (ATD 400, Perkin Elmer, Waltham, MA, USA). Primary desorption was carried out at 250 °C (15 min) to a cold trap (30 mg Tenax TA, 5 °C), with a helium flow of 50 mL min^−1^. Volatiles were desorbed from the cold trap to the GC-column by heating to 300 °C for 4 min (secondary desorption), using a split ratio of 1:10. The volatiles were transferred through a heated transfer-line (225 °C) to a gas chromatograph/mass spectrometer (GC–MS, 7890A GC-system interfaced with a 5975C VL MSD (Mass Selective Detector) with Triple-Axis detector from Agilent Technologies, Palo Alto, Santa Clara, CA, USA) equipped with a J & W Scientific DB-Wax column (30 m × 0.25 mm × 0.25 µm) using helium as carrier gas (1 mL min^−1^). The column temperature was kept at 40 °C for 10 min, increased with 8 °C min^−1^ to 240 °C, and kept isothermal for 5 min. The mass selective detector used the electron ionization mode, and the mass/charge (*m/z*) range between 15 and 300 was scanned.

The software program, MSD-Chemstation (Version E.02.00, Agilent Technologies, Palo Alto, California), was used for data analysis, and the volatile compounds were identified by probability-based matching with mass spectra in the G1035A Wiley library (Hewlett-Packard, Palo Alto, Santa Clara, CA). Calculations were based on peak areas divided by area of internal standard (arbitrary unit based on single ions selected for each individual compound).

### 2.4. Sensory Evaluation

Simplified descriptive sensory evaluations were performed by five subjects belonging to the project group. During two training sessions, the panel developed and trained six sensory descriptors, i.e., ‘color’ (in practice degree of yellow/brown color), ‘overall odor’, ‘apple flavor’, ‘overall flavor’, ‘sweet taste’ and ‘sour taste’. A reference juice (cultivar ‘Jonagored’) was used for training the descriptors; and for sweet and sour taste, sucrose (11%) and malic acid (0.5%) solutions in water were also used; 50 mL of juice was served at room temperature in clear wine glasses with glass petri dishes as lids. The attribute intensities of the samples were evaluated on a 15 cm unstructured continuous line-scale, ranging from 0 (none) to 14 (very much), and the scores of each sample were averaged over five assessors. For the assessors to memorize the intensity of the descriptors between sensory evaluation sessions, a reference juice of cultivar Jonagored with its intensities pre-scored at the evaluation score sheets were used as the calibration sample at all sessions. Samples were served in random order, and at each session twenty juices were evaluated, with a short break after every four samples. Crackers, fresh cucumber and water were provided during the sensory evaluation sessions. Due to the large number of samples and a limited sample amount available, each juice was evaluated once by each judge. Moreover, distinct odors (e.g., ‘artichoke’) and flavors (e.g., ‘pear’, ‘peach’, ‘pineapple’ and ‘citrus’) were identified by the judges for some of the tested juices. In particular, the flavors listed in “The World’s first flavor wheel for apples” [12], were used to help the panel group in the juice description.

### 2.5. Bruker Spin Generated Fingerprint (SGF) Fruit Juice Screener

Bruker Spin Generated Fingerprint (SGF) profiling [13,14] was employed for the NMR analysis. Each sample required minimal preparation consisting of 90% juice with 10% buffer containing 0.1% of TSP (sodium salt of 3-trimethylsilyl-propionate acid-d_4_) and 0.013% of sodium azide to suppress microbial activity. This NMR-based screening method is based on an Avance 400 NMR spectrometer with a 9.4-T Ultrashield™ Plus magnet and utilizes flow-injection NMR (BEST™ NMR) with a 4-mm flow-cell probe with Z-gradient and a Gilson liquid handler for sample storage, preparation and transfer. Samples were provided in bar-coded cryo-vials placed in a Gilson cooling rack that kept the temperature low (about 4 °C) prior to injection. Then a heated transfer line from the Gilson unit to the probe allowed the pre-equilibration of the sample to the desired temperature (300 K) during the transfer. The overall experimental procedure was fully controlled by Bruker’s SampleTrack™ software including temperature adjustment, tuning and matching, locking, shimming and the optimization of the pulses and presaturation power for each sample. Two NMR experiments were executed: the pulse sequence ‘noesygppr1d’ allowed a quantitative evaluation even close to the water signal while the sequence ‘jresgpprgf’ was employed to obtain a fast 2D J-resolved spectrum [15] which facilitates unambiguous signal identification. The resulting spectra did not need any manual processing step, as they were automatically phase corrected and referenced by the Bruker procedure. 

#### 2.5.1. Quantification (Targeted Analysis)

After the acquisition of spectra, the identification and absolute quantification of 26 compounds was performed by comparing the acquired sample spectra with the reference spectra. The compounds included: sugars (sucrose, glucose, fructose, and xylose), acids (malic acid, citric acid, citramalic, succinic acid, chlorogenic acid, quinic acid) and indicators of apple juice quality (proline, alanine, 5-hydroxymethylfurfural, ethanol, methanol, acetaldehyde, benzaldehyde, acetoine, arbutin, and the acids lactic, fumaric, formic, benzoic, pyruvic, sorbic, and galacturonic). Furthermore, two useful relationships between compound concentrations were calculated (glucose/fructose and malic/quinic acid ratios) as well as the total sugar content for a total of 29 parameters. These are very helpful parameters for the detection of frauds and/or not optimal processing and storage conditions in commercial apple juices. For example, an exhaustive enzymatic treatment of the apple juice is detectable from an increase of galacturonic acid and the usage of unripe fruits is revealed by high concentration of quinic acid while 5-hydroxymethylfurfural is formed by acid-heat catalyzed degradation of sugars and often used as an index for heat, storage, and processing abuse [16]. 

#### 2.5.2. Statistical Analysis (Non-Targeted)

The non-targeted approach consisted of an exhaustive statistical analysis based on a large reference database of 1171 apple juice samples from eight countries (China, Poland, Germany, Turkey, Brazil, Spain, Italy, and Hungary). This analysis allows us to discriminate between direct juice and juice from concentrate and it can detect also the type of fruit and its geographical origin. Then, the sample is verified in two steps. First, a univariate analysis compares each spectral region of interest with the reference data set and detects deviations in compound concentrations. The second approach is a multivariate analysis based on the principal component analysis (PCA)/SIMCA (Soft Independent Modeling of Class Analogy) approach [17] for detecting deviations which are not apparent in a univariate analysis. If both methods give the same inconspicuous result, the sample is consistent with the models.

### 2.6. Multivariate Data Analysis

Four different datasets containing (i) metabolite quantification from SGF profiling, (ii) headspace GC–MS relative peak areas, (iii) ion chromatography quantifications, and (iv) sensory evaluation scores were submitted to PLS toolbox version 8.1.1 (Eigenvector Research, Manson, USA) in the MATLAB (Version 8.6.0.267246, R2015b, The Mathworks, Inc., Natick, MA, USA) environment. Prior to the principal component analysis (PCA) [18], the variables were mean centered and scaled to unit variance. This kind of pre-process, called autoscaling, let all variables have a standard deviation of one and be analyzed on the basis of correlations instead of covariances, as is the case with mean centering [19].

The four datasets were then combined in a single block of data in order to consider the correlations among the different analytical parameters together with the exploration of similarities and differences among the juices analyzed.

The raw 1D NOESY NMR spectra provided by Bruker were also submitted to multivariate data analysis. However, they were first imported in Matlab using an in-house developed routine, aligned using the interval correlation optimized shifting algorithm ‘icoshift’ [20], and then normalized using an artificially generated signal at 12 ppm. The presence of this artificial standard signal allows the scaling of the signal intensity according to changes in parameters and experimental conditions [21]. The spectra were then mean centered. The assignment of the major and minor components of apple juice was carried out according to literature references [22,23,24,25].

## 3. Results and Discussion

### 3.1. Sugar and Acid Measurements

The results obtained by ion chromatography and refractometer are presented in Table 2 and in Supplementary Table S1. The average level of °Brix in the apple juices was 11.4 ranging from 8.1–14.2. With an average of 54.4 g L^−1^, ranging from 30.1 to 78.3 g L^−1^ fructose was the component found in the highest amount, in agreement with the literature [26,27,28]. Both the glucose and sucrose concentrations were rather variable, ranging from 5.4 to 20.7 (average 12.0) g L^−1^ and from 8.5 to 63.2 (average 33.5) g L^−1^, respectively. Sucrose is higher and glucose lower than reported by Eisele and Drake [28], who found averages of 21.6 g L^−1^ sucrose and 20.1 g L^−1^ glucose in juices from 175 apple cultivars grown in 12 countries and different regions in the USA. Also the dominant acid in apples, malic acid, was found in concentrations in agreement with literature values. (8.8 g L^−1^, versus 8.5 g L^−1^ in juices from apples grown in 12 countries and different regions in the USA [28] and 7.4 g L^−1^ in juices obtained from Polish/French apple cultivars [29]). However, the range was remarkably wide with values from 2.2–18.9 g L^−1^. The average citric acid concentration of 0.104 g L^−1^ was also similar to what has been reported earlier [28,29,30]. In the present study, the average succinic acid level was found to be 6.4 mg L^−1^, with non-detectable levels in many cultivars. Averages of 15 mg L^−1^ [31] down to 0.06 mg L^−1^ succinic acid [32] have earlier been reported in apples. Also, the sugar/acid ratio varied widely, from 5.8 to 33.1 with an average of 12.0. Sugar/acid ratios at 15–16 have been shown to give desired sweet taste-sourness balance of juices of Danish apple cultivars [33]. Thus, as expected in a cool climate as Denmark, the average apple juice is in the slightly sour range, but due to the large range in especially malic acid, unique cultivars were also identified with very high sugar/acid ratios (e.g. ‘Søde æbler fra Alsrode’).

**Figure 2:**
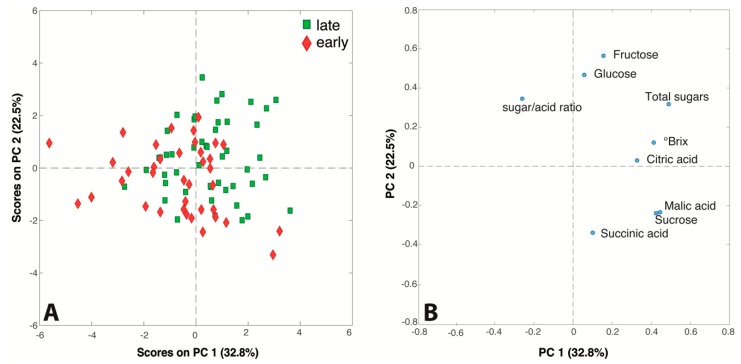
(**A**) Score and (**B**) loading plots of the PCA model performed on the ion chromatography and °Brix dataset.

The data matrix obtained by ion chromatography and °Brix analysis was also submitted to PCA to detect trends and correlations among variables and samples. The PC1/PC2 score and loading plots (55.3% of explained variance) generated by the model are shown in Figure 2. The samples in the score plot have been colored according to the harvest season of the respective apple cultivars: a slight trend is detectable along the diagonal of the plot, since all the late-season juices seem to gather in the top right area while the late-season juices lie mostly in the bottom left part. 

The variables responsible for this trend are the sugars (fructose, glucose, total sugar and °Brix value), suggesting, as expected, that juices produced from late season cultivars allow high-content sugar products to be obtained, while early season apples are suitable for juices low in sugar. Interestingly, the loading plot shows the correlations among the measured variables. Malic acid and sucrose, which are very close in the bottom of the plot, are positively correlated, and both are negatively correlated with fructose and glucose that lie in the top part of the plot. The inverse relationship between malate accumulation and sugars (glucose and fructose) formation in apples has been previously reported [34,35]. The reason of this phenomenon has been attributed to the transformation of malate into sugars via gluconeogenesis during fruit development [35]. At the same time, the converse levels of sucrose and glucose may be due to their interconversion while fructose is mainly formed from sorbitol and is much more highly abundant in apples than glucose [23,36].

### 3.2. Dynamic Headspace Gas Chromatography/Mass Spectrometry (GC–MS)

**Figure 3:**
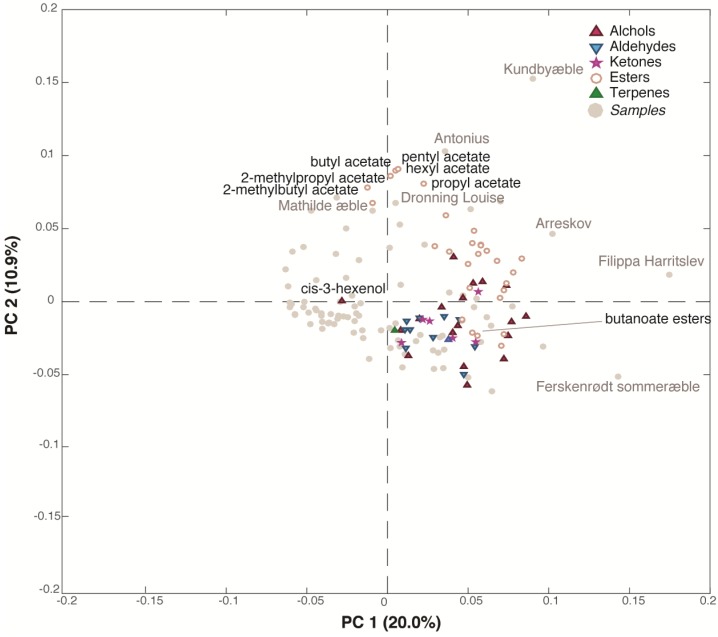
Bi-plot of the PCA model performed on the dynamic headspace GC-MS dataset.

A total of 65 aroma compounds were identified in the juices, the main chemical groups being esters, alcohols, aldehydes and ketones (Supplementary Table S2) as previously reported [37]. This dataset (86 samples x 65 variables) was submitted to PCA, and it showed large differences between aroma profiles of the juices. The PC1/PC2 (30.9% of explained variance) bi-plot (Figure 3), which shows both the samples and the variables distribution, indicates that the majority of the juice samples are situated in the bottom left part of the plot, in the opposite direction of the variables. This means that they are characterized by low values of all the volatile compounds except for cis-3-hexenol. Indeed, all the alcohols (except cis-3-hexenol), esters, ketones, aldehydes lay in the right side of the plot, having high loadings values on PC1, while a small group of acetate esters (pentyl, hexyl, propyl, butyl, 2-methylpropyl and 2-methylbutyl acetate) is grouped at the top of the plot (high loadings values on PC2). The samples were found to be richer in aroma compounds (very right region of the plot) were ‘Arreskov’, ‘Filippa Harritslev’ and ‘Ferskenrødt sommeræble’ while the juices produced from ‘Kundbyæble’, ‘Antonius’, ‘Dronning Louise’ and ‘Mathilde æble’ showed a high content of acetate esters being situated in the top of the plot. Interestingly, the butanoate esters tended to gather with alcohols, aldehydes and ketones along PC1 together with hexanoate esters. For apples, groupings according to acetate and/or butanoate esters have been reported earlier [38,39]. The demonstrated correlation within the different acetate esters and butanoate esters, respectively, could indicate common formation pathways and genetic background.

### 3.3. Sensory Evaluation

**Figure 4:**
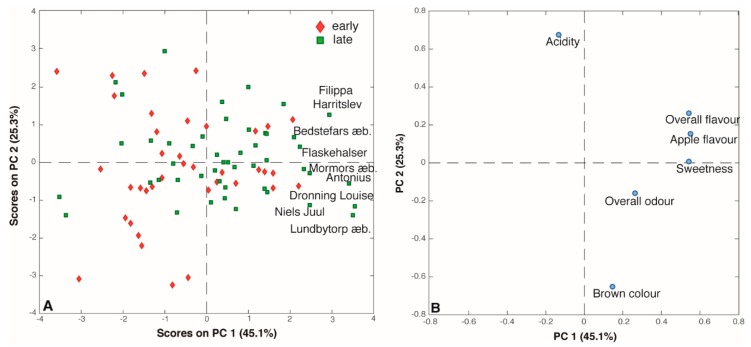
(**A**) Score and (**B**) loading plots of the PCA performed on the sensory analysis dataset.

Six sensory attributes were evaluated by the assessors: sweet taste, sour taste, brown color, apple flavor, overall flavor and overall odor. Thus, the scores obtained by the panel test generated an 86 x 6 matrix that was submitted to PCA. The PC1/PC2 (70.4% of total variance) score plot reported in Figure 4A shows that, as seen for the aroma compounds, there is no clear grouping according the harvest period of the apples. Interestingly, the loading plot (Figure 4B) helps to understand the correlations among the six sensory descriptors. Indeed, ‘overall flavor’, ‘apple flavor’ and ‘sweet taste’ appear to be strictly correlated, being well clustered on the right part of the plot (high PC1 loadings), while sour taste and brown color seem to be inversely correlated given the fact that they are situated in the opposite direction of the PC2. Thus, the most appreciated juices, according to the scores, were the ones obtained from ‘Filippa Harritslev’, Bedstefars æble’, ‘Flaskehalser’, ‘Mormors æble’, ‘Antonius’, ‘Dronning Louise’, ‘Niels Juul’, and ‘Lundbytorp æble’. Seven of these eight samples were also given distinct odors and flavor. Indeed, fresh green odor and fresh apple flavor were assigned to ‘Filippa Harritslev’ and ‘Bedstefars æble’ juices while the odor and flavor of the ‘Mormors æble’s juice resemble apricots. Interestingly, the juice obtained from the ‘Antonius’ cultivar turned out to have a peach-like odor and a flavor reminiscent of tropical fruits. Overall, the sensory panel noted distinct odor notes for 31 out of 86 of the juices, while 56 juices were characterized by peculiar flavors. Most of the odors resembled other fruits like ‘pear’, ‘peach’, ‘pineapple’ and ‘berries’, but also ‘Rhubarb’ was identified and in one juice (‘Auroravej’) turned out to recall ‘artichoke’ flavor (Supplementary Table S3). 

### 3.4. Bruker-SGF Fruit Juice Screener

#### 3.4.1. Metabolite Quantification

**Figure 5:**
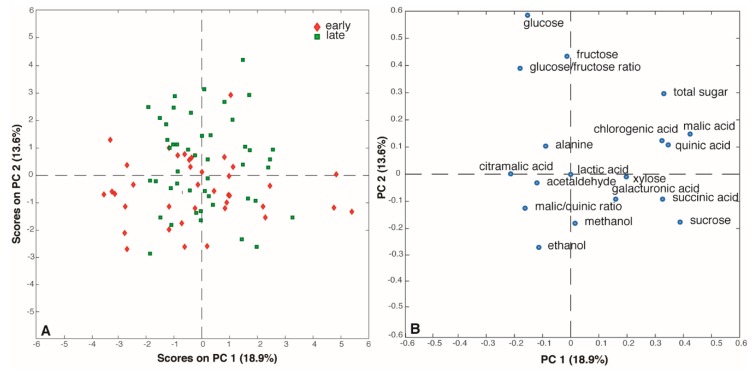
(**A**) Score and (**B**) loading plots of the PCA performed on the Bruker SGF profiling dataset.

The output of the quantification analysis performed by the Bruker-SGF profiling is reported in Supplementary Table S4. Sugar and acid concentrations were in agreement with the ion chromatography measurements reported in Table 2. Interestingly, average sucrose levels turned out to be slightly above the maximum value established by the European Fruit Juice Association (AIJN) [40], while average glucose concentration is just below the minimum suggested by the association. Among the 17 indicators of apple juice quality, 12 had, as expected, levels below the limit of quantification (LOQ) of the instrument in all the analyzed samples. However, acetaldehyde, alanine, ethanol, galacturonic acid and methanol could be measured in a few juice samples. In particular, one sample (juice from cultivar ‘Louisendal’) was characterized by acetaldehyde values higher than LOQ (5 mg L^−1^). Alanine was quantified in 69 out of 86 samples and its average concentration was within the range established by AIJN (from 1 to 50 mg L^−1^), except in four samples (juices from the cultivars ‘Skovfoged æble’, ‘Ejby æble’, ‘Gråsten Rød’, and ‘Alsisk Citronæble’) that showed an alanine content above 50 mg L^−1^. A concentration of galacturonic acid higher than 100 mg L^−1^ (LOQ) was detected in ‘Gadeskovæble’ and ‘Mormors æble’ juice samples, and methanol levels above 10 mg L^−1^ (LOQ) was were found in 21 juice samples; this is probably due to its release from methyl esterified apple pectin during the postharvest storage of the apples, as reported in literature [41,42]. Finally, an average ethanol level of 88 mg L^−1^ was detected in 34 samples, in agreement with AIJN recommendations. In order to better explore this dataset, a PCA was performed after filtering out the 12 variables with levels below the LOQ in all samples, as well as the acetaldehyde; thus, only 16 parameters (variables) were included. The PC1/PC2 (32.5% of total variance) score and loading plots are reported in Figure 5. Interestingly, the loading plot indicates a strong correlation among malic acid and two of the minor acids, chlorogenic and quinic acid, that appear very close to each other in the right side of the loading plot (Figure 5B). By contrast, glucose is situated in the opposite direction of sucrose in agreement with the results obtained by ion chromatography.

#### 3.4.2. Raw Nuclear Magnetic Resonance (NMR) Spectra Analysis

**Figure 6:**
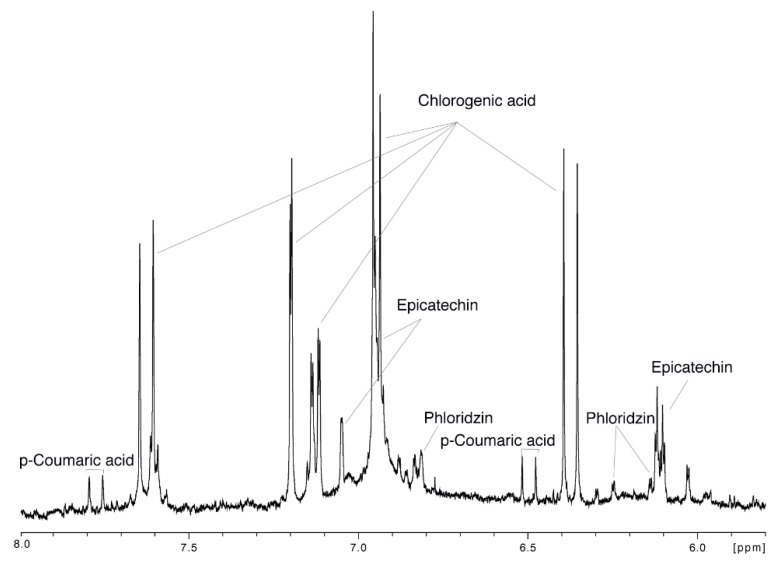
Aromatic region and relative signal assignment of a representative ^1^H-NMR (nuclear magnetic resonance ) spectrum of apple juice.

Only the region between 5.8 and 8.0 ppm of the ^1^H-NMR spectra of the 86 apple juices was considered during the analysis in order to i) remove the spectral region containing the most dominant signals of sugars and acids ii) explore the polyphenols-related variation in the analyzed apple juices. The signal assignment of the aromatic region is reported in Figure 6**,** while the remaining part of the spectrum is showed in Supplementary Figure S1. 

**Figure 7:**
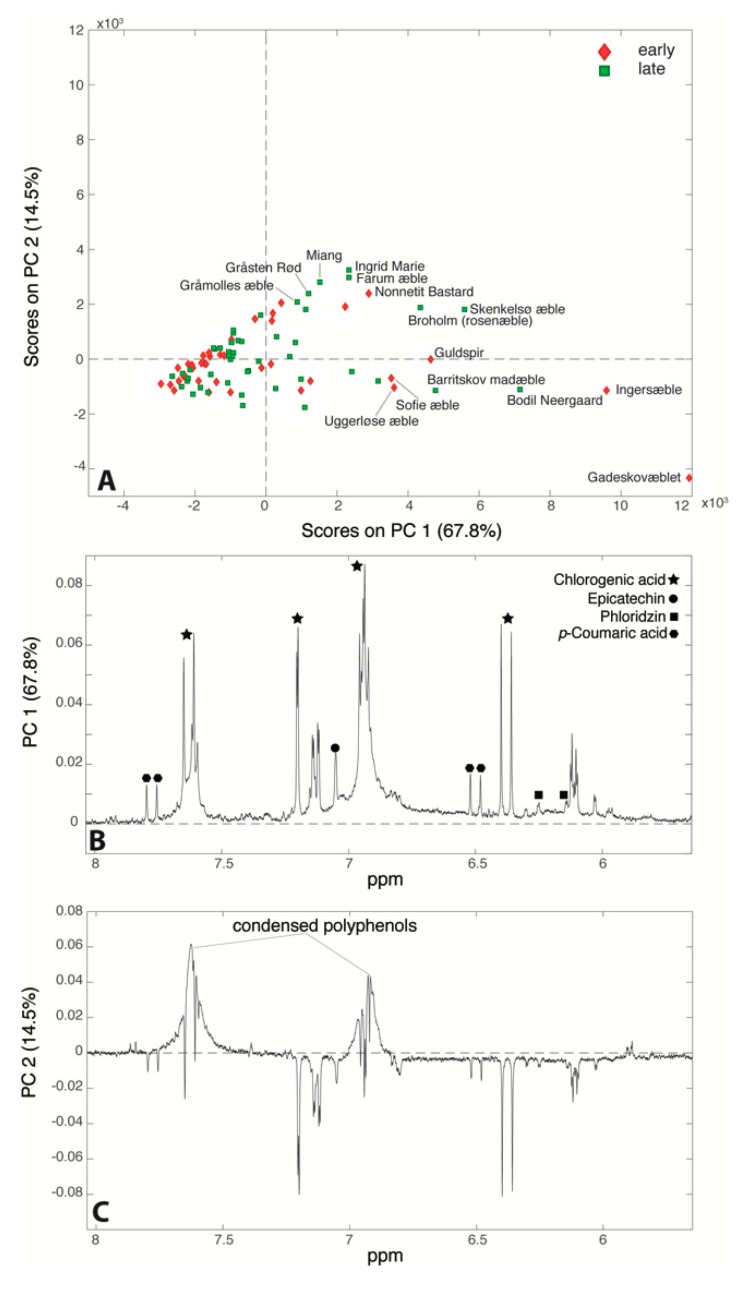
Score plot (**A**) and PC1 (**B**) and PC2 (**C**) loading plots of the principal component analysis (PCA) performed on the ^1^H-NMR spectra of the 86 apple juices under study.

Besides the chlorogenic acid that was already quantified by Bruker, three more polyphenols were identified: epicatechin, phloridzin and *p*-coumaric acid. A PCA (82.3% of total variance) was performed on the 86 spectra, and the relative score and loading plots are reported in Figure 7. Figure 7A clearly shows that juices from cultivars ‘Gadeskovæble’, ‘Ingersæble’, ‘Bodil Neergaard’, and ‘Barritskov Madæble’ (far right region of the plot) are characterized by intense signals of all the polyphenols identified in the NMR spectrum, while the majority of the samples lie in the left part suggesting that their polyphenol content is too low to be detectable by NMR. The PC2 loading plot (Figure 7C) indicates that the samples that are situated in the top of the score plot (‘Ingrid Marie’, ‘Farum æble’, ‘Miang’, ‘Gråsten rød’ and ‘Nonnetit Bastard’) are characterized by higher intensities of condensed polyphenols signals (around 6.9 and 7.7 ppm): the aggregation of monomeric phenolic compounds (epicatechin, chlorogenic acid, phloridzin and p-coumaric acid) is probably mainly due to non-enzymatic oxygenation as reported elsewhere [22,23]. At the same time, the samples that lie at the bottom of the score plot only present the sharp signals of the monomeric polyphenols (chlorogenic acid, epicatechin, phloridzin and *p*-coumaric acid), which also suggests a strong correlation among these compounds. Vermathen et al. also found that chlorogenic acid and epicatechin were positively correlated [23]. This correlation may be explained by the shared initial biosynthetic pathway steps of these phenolic compounds both having coumaric acid as common precursor [43]. In a study conducted recently by Kschonsek et al., it was highlighted that apple cultivars such as ‘Braeburn’, ‘Elstar’, and ‘Jonagold’ have a lower content of polyphenols compared to the old cultivars. This is obtained on purpose during the breeding, because of the astringent taste and rapid enzymatic browning given by the polyphenols [9]. Indeed, apple juices obtained from ‘Broholm rosenæble’, ‘Guldspir’, ‘Sofie æble’ (right part of the score plot—high polyphenol content) were assigned a bitter taste as reported in Supplementary Table S3.

### 3.5. Multivariate Analysis on Datasets Combined (Ion Chromatography, Headspace GC–MS, Sensory Evaluation, Bruker-SGF Profiling)

**Figure 8:**
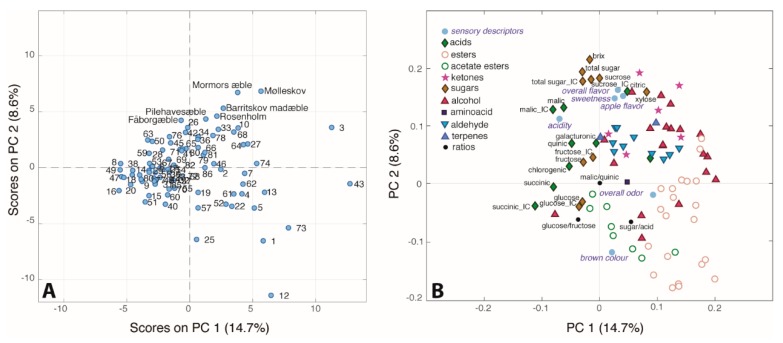
(**A**) Score and (**B**) loading plots of the PCA performed on the 86 × 96 dataset.

The unsupervised chemometric approach PCA was employed to analyse the four different datasets (ion chromatography, headspace GC–MS, sensory evaluation and Bruker-SGF profiling) combined giving a final data matrix of 86 samples and 96 variables. The PC1/PC2 (23.3% of total variance) score plot reported in Figure 8A shows large differences between the juices of different apple cultivars. Many samples lie on the left side of the score plot indicating that juices of these cultivars are characterized by low levels of most of the measured variables. Fewer cultivars are characterized by high levels of many of the variables. Indeed, the loading plot (Figure 8B), shows that the majority of the variables lie on the right side of the plot. Interestingly, the loading plot shows the clustering of all the esters in the right bottom region of the plot, while the alchols and the aldehydes are grouped in the top right part. Finally acids and sugars are situated in the left part of the plot. In particular, sucrose is situated at the top of the score plot together with the °Brix value and total sugar content, all highly correlated with sweet taste, apple flavor and overall flavor. In the opposite direction, we can find glucose, the glucose/fructose ratio, the sugar/acid ratio, the brown colour as well as some esters. The inverse relation between the sucrose and glucose content is already known from literature, and it is likely due to their interconversion [23]. As already reported in a previous work by our group, the sucrose turned out to be the main factor responsible for the sweet taste, even though the fructose is known to be the main sugar and thus sweetener in apple juice [44]. This confirms, once again, the complexity in assigning the sweet taste to a specific chemical compound when it should be considered the result of the synergy of several components [45]. Indeed, the loading plot (Figure 8B) shows that also citric acid, xylose and some ketones and alcohols are correlated with sweet taste and apple flavor. Interestingly, the acetate esters seem to be positively correlated with the brown colour of the apple juice, in agreement with the literature [46]. In turn, brown colour is negatively correlated with the acid content, as shown also in the correlation map (Supplementary Figure S2). The overall odor is mostly correlated with butanoate esters. Moreover, the sour taste of the juices seem as one would expect mainly due to the content of the dominant malic acid.

Finally, the supervised chemometric tool PLS-DA was also employed, in order to detect the variables responsible for the differences between juices obtained from early and late season cultivars. The intensity of the sensory descriptors ‘apple flavor’, ‘overall flavor’ and ‘sweet taste’ were higher in juices of apples harvested late in the season compared to early in the season, as well as glucose, total sugar content and °Brix values (Supplementary Figure S3). By contrast, 2-hexen-1-ol, cis-3-hexenol, hexanal and 3-methyl-3-butenol were present at the highest levels in the early cultivars juices. Most aldehydes also had highest levels in early-season juices. 

It has to be noted that many factors in the growing conditions can influence the fruit quality, and it seems to be a general tendency to obtain better development of aroma and colour when ripening is happening in a cool climate with cool nights [47,48]. Moreover, both juice constituents and sensory characteristics can be influenced by other parameters than cultivars, such as climate, soil, and processing methods [46,49]. However, we kept these variables constant here, since all the cultivars were harvested in the same year, from the same orchard and processed in the exact same way. Also fruit/leaf ratio strongly influences fruit development and quality, and all trees were handthinned in early season to ensure a good fruit/leaf ratio and thus facilitate optimal fruit development [50]. Also fruit maturity and ripeness are fundamental for the juice characteristics [47,51], for this reason, a team of experts decided the optimal picking and post-harvest storage time for each cultivar.

## 4. Conclusions

In this work, juices obtained from 86 apple cultivars, of which most are ancient Danish cultivars, have been extensively analyzed for the first time by employing traditional (ion chromatography, dynamic headspace, sensory evaluation) and cutting-edge NMR technology (Bruker-SGF profiling). Large variations with respect to sugars, acids, aroma compounds and sensory attributes were observed among the different samples. The chemical composition of all the analyzed juices were within the general reported ranges of apple juice, however, four cultivars (‘Gadeskovæble’, ‘Ingersæble’, ‘Bodil Neergaard’, and ‘Barritskov Madæble’) yielded juices particularly rich in polyphenols, while ‘Mormors æble’ and ‘Antonius’ juices were characterized by very peculiar odors and flavors such as apricot and peach, respectively. Moreover, we observed the tendency for late-season juices to be characterized by higher °Brix values and sugar content, which is linked to a sweeter and more flavor intense profile than early-season juices. The data reported here could be used for the production of specialty single cultivar ‘vintage’ apple juices (i.e., to be used as juice menus at restaurants), or mixed juices to obtain a final product that is characterized both by healthy properties and peculiar odors and flavors.

## Figures and Tables

**Table 1:** Information about the 86 cultivars collected *^a^*.

Sample	Apple Variety	Country of Origin	Usage	Introduction Date	Harvest Season *^b^*	Picking Day *^c^*
1	Louisendal	Unknown	unknown	Unknown	E	1
2	Skovfoged	Denmark	Dessert	1830	E	2
3	Ferskenrødt sommeræble	France	Dessert	before 1839	E	2
4	Ejby æble	Denmark	Dessert	1840	E	5
5	Augustæble	Netherlands	dessert, culinary	before 1795	E	5
6	Uggerløse æble	Unknown	unknown	Unknown	E	9
7	Herschendsgave	Denmark	Dessert	1850	E	16
8	Gadeskovæble	Denmark	Dessert	1924	E	19
9	Auroravej	Unknown	unknown	Unknown	E	19
10	Ondrup sommeræble	Denmark	Dessert	1900	E	16
11	Fåborgæble	Denmark	Culinary	Unknown	E	29
12	Kundbyæble	Denmark	Dessert	1995	E	29
13	Ingers æble	Denmark	Dessert	1870	E	29
14	Ørdings æble	Denmark	culinary	Unknown	E	30
15	Ulderup æble	Denmark	Dessert	1890	E	29
16	Thyregod kalvil	Denmark	Dessert	1800	E	29
17	Fuhræble	Norway	unknown	1660	E	29
18	Guldspir	Denmark	culinary	1937	E	29
19	Gravenfin	Denmark	Dessert	1932	E	29
20	Sofie æble	Denmark	Dessert	1896	E	29
21	Nina’s æble	Denmark	Dessert	1835	E	29
22	Søde æbler fra Alsrode	Denmark	Dessert	Unknown	E	29
23	Vallekilde Sommeræble	Denmark	Dessert	1913	E	29
24	Rosenholm	Denmark	culinary	1870	E	19
25	Dynnegårdsæble	Denmark	dessert, culinary	before 1924	E	29
26	Nonnetit Bastard	Denmark	dessert, culinary	1800	E	29
27	Miang æble	Denmark	Dessert	before 1913	L	51
28	Gråsten gul	Denmark	dessert, cider, culinary	1750	L	39
29	Pigeon Stribet	Denmark	Dessert	about 1860	L	52
30	Butteræble	Unknown	unknown	Unknown	E	29
31	Vejløæble	Denmark	unknown	Unknown	E	29
32	Nybøllegård	Denmark	unknown	Unknown	E	29
33	Fejø æble	Denmark	Dessert	1913	E	29
34	Hindbæræble	Denmark	culinary	1876	E	29
35	Pilehavesæble	Denmark	culinary	1800	L	39
36	Langt rødt Hinbæræble	Germany	dessert, culinary	before 1802	E	29
37	Mosede æble	Unknown	unknown	Unknown	E	29
38	Ingrid Marie	Denmark	dessert, culinary, cider	1910	L	50
39	Maglemer rød	Denmark	Dessert	Unknown	E	29
40	Fynsk udvalg V	Denmark	Dessert	1960’ies	E	29
41	Lundbytorp æble	Denmark	dessert, culinary	1913	L	50
42	Bodil Neergård	Denmark	dessert, culinary	1850	L	50
43	Filippa Harritslev	Denmark	dessert	Unknown	L	50
44	Tønnes	Denmark	dessert, cider, culinary	1820	L	50
45	Flaskehalser	Denmark	dessert, culinary	before 1913	L	50
46	Flintinge	Denmark	cullinary	before 1889	L	50
47	Jakober	Denmark	culinary	before 1850	L	39
48	Æbeltoftæble	Denmark	culinary	Unknown	L	43
49	Skenkelsø æble	Denmark	culinary	1792	L	50
50	Broholm Rosenæble	Denmark	dessert	1866	L	50
51	Ondrup moseæble	Denmark	dessert, culinary	1850	E	29
52	Høje Taastrup æble	Denmark	dessert	Unknown	E	17
53	Knud Lunn	Denmark	culinary	1865	L	50
54	Niels Juul	Denmark	dessert, culinary	1875	L	50
55	Thyrislund	Denmark	dessert	Unknown	L	50
56	Broholm	Denmark	dessert	1866	L	50
57	Skensved æble	Denmark	dessert	1800	E	8
58	Pigeon spejlsby	Denmark	dessert	1850	L	52
59	Borgherre	Netherlands	culinary	before 1788	L	50
60	Mathilde æble	Denmark	dessert	1913	L	52
61	Jølbyæble	Denmark	culinary	1938	L	40
62	Gråsten rød	Germany	dessert, cider, culinary	before 1875	L	45
63	Holstenhus	Denmark	dessert, culinary	1875	L	40
64	Nonnetit fra Als	Denmark	dessert	Unknown	L	39
65	Pigeon Rød Vinter	Denmark	culinary	Unknown	L	39
66	Pigeon fra Maribo	Denmark	dessert	1930	L	39
67	Alsisk Citronæble	Denmark	culinary	before 1911	L	37
68	Antonius	Denmark	dessert, culinary	1932	L	37
69	Fynsk udvalg II	Denmark	dessert, culinary	1960’ies	L	38
70	Annas æble	Denmark	dessert	1900	L	37
71	Bedstefars æble	Denmark	dessert	Unknown	L	37
72	Elstar	Netherlands	dessert	1955	L	37
73	Arreskov	Denmark	dessert	1850	L	38
74	Gråsten Høvdinggård	Denmark	dessert, culinary	1927	L	38
75	Barritskov madæble	Denmark	dessert, culinary	1890	L	44
76	Gråmølles æble	England	culinary	1740	L	44
77	Farum æble	Denmark	culinary	1900	L	44
78	Ildrød Pigeon	Denmark	dessert	1800	L	44
79	Dronning Louise	Denmark	dessert	1892	L	45
80	Risskov Rambour	Denmark	culinary	1870	L	50
81	Tagesminde æble	Denmark	culinary	Unknown	L	45
82	Apple 207 Knuthenborg	Denmark	culinary	Unknown	L	46
83	Mormors æble	Denmark	dessert	Unknown	L	45
84	Mølleskov	Denmark	dessert, culinary	1840	L	37
85	Herfølge voksæble	Denmark	dessert, culinary	1825	L	45
86	Lise Legind	Denmark	dessert	1880	L	45

*^a^* Data about country of origin, usage and introduction date have been compiled from the ‘Pometum apple key’ database. *^b^* Harvest season has been classified in E = early and L = late. *^c^* Picking day 1 corresponds to 23 August while day 52 corresponds to 14 October.

**Table 2:** Average concentrations of sugars and acids in the 86 apple juices, determined by ion chromatography.

	Units	Mean	SD *^a^*	% CV *^b^*	Min	Max	Range
°Brix	%	11.4	1.2	10.5	8.1	14.2	6.1
Sucrose	g L^−1^	33.5	11.6	34.7	8.5	63.2	54.8
Glucose	g L^−1^	12.0	3.9	32.7	5.4	20.7	15.3
Fructose	g L^−1^	54.4	9.3	17.1	30.1	78.3	48.3
Total sugar	g L^−1^	100.0	13.5	13.5	63.2	134.4	71.2
Malic	g L^−1^	8.80	2.46	28.0	2.2	18.9	16.7
Citric	g L^−1^	0.104	0.060	57.8	0.000	0.308	0.308
Succinic	g L^−1^	0.006	0.006	100.8	0.000	0.033	0.033
Sugar/acid ratio		12.0	3.5	29.1	5.8	33.1	27.3

*^a^* SD = standard deviation. *^b^* % CV = percent coefficient of variation calculated as (SD/mean) *100.

## Supplementary Materials

The following are available online at https://www.mdpi.com/2218-1989/9/7/139/s1, ‘Analytical platforms reliability’. Figure S1: Signal assignment of a representative ^1^H-NMR spectrum of apple juice (0–5.5 ppm). Figure S2: Correlation map generated from the 86 x 96 matrix. The variables are grouped by similarity. Figure S3: (A) LV1/LV2 scores plot of the PLS-DA model developed to discriminate early-season juices (class 1) from late (class 2) harvest cultivars. Most discriminative markers are shown in the regression vector plot (C) and VIP score plot (D). Area Under the Curve (AUC) of Receiver Operating Characteristic (ROC) (E) and sensitivity and specificity (F) plot of the PLS-DA model. Table S1: °Brix values of the 86 juices., Table S2: List of 65 compounds identified by GC-MS analysis. Table S3: Distinct odors and flavors of the 86 juices and numerical evaluation of their sensory attributes. Table S4: Average concentrations of the compounds and parameters identified from Bruker SGF profiling.
