# Supplementary material for: Ancient Danish Apple Cultivars—A Comprehensive Metabolite and Sensory Profiling of Apple Juices
Source: Metabolites. 2019 Jul 11;9(7):139. doi: 10.3390/metabo9070139 (PMC6680383; doi:10.3390/metabo9070139)
Supplement: Supplementary file 1 [file metabolites-09-00139-s001.pdf]

Supplementary Material

Ancient Danish apple cultivars – A comprehensive metabolite and sensory profiling of apple juices

Nunzia Iaccarino [1], Camilla Varming [2], Mikael Agerlin Petersen [3], Nanna Viereck [3], Birk Schütz [4], Torben Bo Toldam-Andersen [5], Antonio Randazzo [1], and Søren Balling Engelsen [3,*]

[1] Department of Pharmacy, University of Naples Federico II, Via D. Montesano 49, 80131 Naples, Italy; nunzia.iaccarino@unina.it
[2] Novozymes, Biologiens Vej 2, 2800 Kgs. Lyngby, Denmark
[3] Department of Food Science, University of Copenhagen, Rolighedsvej 26, 1958 Frederiksberg C, Denmark
[4] Bruker BioSpin, Silberstreifen 4, 76287 Rheinstetten, Germany
[5] Department of Plant and Environmental Sciences, University of Copenhagen, Hoejbakkegaard Alle 13, 2630 Taastrup, Denmark

* Correspondence: se@food.ku.dk; Tel.: +45 20 20 00 64 (S.B.E.)

Table of Contents

No.	Content	Page
1	**Analytical platforms' reliability**	S3
2	**Table S1.** °Brix values of the 86 juices.	S5
3	**Table S2.** List of 65 compounds identified by GC-MS analysis.	S7
4	**Table S3.** Distinct odors and flavors of the 86 juices and numerical evaluation of their sensory attributes.	S9
5	**Table S4.** Average concentrations of the compounds and parameters identified from Bruker SGF profiling.	S11
6	**Figure S1.** Signal assignment of a representative ^{1}H-NMR spectrum of apple juice (0 – 5.5 ppm).	S12
7	**Figure S2.** Correlation map generated from the 86 x 96 matrix. The variables are grouped by similarity.	S13
8	**Figure S3.** (A) LV1/LV2 scores plot of the PLS-DA model developed to discriminate early-season juices (class 1) from late (class 2) harvest cultivars. Most discriminative markers are shown in the regression vector plot (C) and VIP score plot (D). Area Under the Curve (AUC) of Receiver Operating Characteristic (ROC) (E) and sensitivity and specificity (F) plot of the PLS-DA model.	S14

Analytical platforms' reliability

NMR

This study focuses on the employment of a recently developed NMR-based juice screener (SGF profiling) for the analysis of juices obtained by ancient Danish apple cultivars. It is known that two NMR determinations give the same results (large metabolomic studies usually employ single determination); this is why only one measurement was performed in this work. Even though two independent preparations of the juices would have better described the intra-juice variability, our study was more oriented toward describing the inter-apple juice variability rather than describing each apple cultivar in detail.

Ion Chromatography (IC)

To measure IC accuracy, a Quality Control sample was prepared by mixing five standards (citric acid, malic acid, glucose, fructose and glucose) and it was run seven times. The areas under the peak corresponding to each molecule together with their standard deviation, average and percent coefficient of variation (%CV) across the seven replicates, are reported in the table below. The reported values of %CV (less than 2%) confirm the reliability of the IC platform employed in our study.

	**Citric acid**	**Malic acid**	**Glucose**	**Fructose**	**Sucrose**
Standard mix_1	10.619	41.143	7028.728	11763.45	3505.707
Standard mix_2	10.484	41.032	6976.936	11575.77	3493.564
Standard mix _3	10.669	41.35	7028.266	11603.51	3578.934
Standard mix _4	10.544	41.296	7017.829	11575.37	3598.164
Standard mix _5	10.284	40.249	6759.458	11312.84	3451.197
Standard mix _6	10.362	40.278	6767.272	11318.27	3454.808
Standard mix _7	10.358	40.279	6751.946	11307.38	3439.516
STANDARD DEVIATION	0.1449997	0.5111117	136.6152	180.9388	63.18577
AVERAGE	10.474286	40.803857	6904.348	11493.8	3503.127
CV[a]	0.0138434	0.0125261	0.019787	0.015742	0.018037
%CV	1.38	1.25	1.98	1.57	1.80

[a]CV = Coefficient of Variation calculated on the 7 replicates.

Gas Chromatography/Mass Spectrometry (GC-MS)

The accuracy of the analysis of aroma compounds by dynamic headspace GC–MS was already tested in our laboratory on a different apple juice dataset consisting of seventeen samples analyzed in quadruplicate. The %CV for each aroma compound, across the four replicates, is reported in table below. For most compounds, the %CV was between 0.2 and 15%. For few compounds the values were higher, mainly due the compounds being present in very small concentrations.

Aroma compound	%CV[a]
Alcohols	
2-methyl-1-butanol	0.2
2-methyl-1-propanol	0.5
2-ethyl-1-hexanol	4.2
2-hexen-1-ol	1.3
3-methyl-3-butenol	4.9
cis-3-hexenol	5.4
6-Methyl-5-hepten-2-ol	6.3
heptanol	1.5
hexanol	0.2
octanol	1.3
propanol	0.7
butanol	0.2
Esters	
2-methylbutyl acetate	0.8
2-methylpropyl acetate	3.3
hexyl acetate	0.7
pentyl acetate	2.3
propyl acetate	2.7
ethyl acetate	55.0
butyl acetate	0.5
methyl acetate	46.5
propyl 2-methylbutanoate	2.1
butyl butanoate	0.9
hexyl butanoate	2.3
methyl 2-methylbutanoate	3.8
butyl 2-methyl butanoate	1.6
propyl butanoate	1.0
butyl propanoate	2.7
ethyl 2-methylpropanoate	3.7
ethyl 2-methylbutyrate	0.7
ethyl butyrate	0.3
hexyl-2-methyl butyrate	2.7
methyl butyrate	3.6
methyl hexanoate	16.6
ethyl hexanoate	0.9
butyl hexanoate	3.6
ethyl pentanoate	3.3
ethyl propanoate	32.0
ethyl octanoate	14.3
Aldehydes	
butanal	71.4
heptanal	5.2
hexanal	0.3
nonanal	2.8
octanal	6.3
t-2-hexenal	2.0
Ketons	
2-butanone	38.8
2-heptanone	16.0
Terpenes	
farnesene	9.3

[a]%CV = Percentage coefficient of variation calculated on 4 replicates.

Table S1. °Brix values of the 86 juices.

#	Apple cultivar	°Brix
1	Louisendal	9.7
2	Skovfoged	10.6
3	Ferskenrødt sommeræble	11.1
4	Ejby æble	10.2
5	Augustæble	9.00
6	Uggerløse æble	10.3
7	Herschendsgave	12.3
8	Gadeskovæblet	10.8
9	Auroravej	12.1
10	Ondrup sommeræble	12.8
11	Fåborgæble	12.6
12	Kundbyæble	9.4
13	Ingers æble	10.0
14	Ørdings æble	10.4
15	Ulderup æble	10.4
16	Thyregod kalvil	10.5
17	Fuhræble	11.0
18	Guldspir	10.3
19	Gravenfin	11.9
20	Sofie æble	9.5
21	Nina's æble	11.2
22	Søde æbler fra Aalsrode	8.0
23	Vallekilde Sommeræble	10.5
24	Rosenholm	12.0
25	Dynnegårdsæble	11.2
26	Nonnetit Bastard	13.2
27	Miang æble	10.5
28	Gråsten Gul	11.6
29	Pigeon Stribet	11.8
30	Butteræble	12.3
31	Vejløæble	11.9
32	Nybøllegård	11.4
33	Fejø æble	12.0
34	Hindbæræble	10.1
35	Pilehavesæble	13.2
36	Langt rødt Hinbæræble	11.6
37	Mosede æbler	11.6
38	Ingrid Marie	11.8
39	Maglemer rød	9.2
40	Fynsk udvalg V	9.0
41	Lundbytorp æble	11.6
42	Bodil Neergård	13.5
43	Filippa Harritslev	11.3
44	Tønnes	12.2
45	Flaskehalser	12.6
46	Flintinge	11.9
47	Jakober	11.6
48	Æbletoftæble	10.8
49	Skenkelsø æble	13.3
50	Broholm Rosenæble	13.5
51	Ondrup moseæble	11.3
52	Høje Taastrup æble	9.8
53	Knud Lunn	11.5
54	Niels Juul	11.8
55	Thyrislund	10.6
56	Broholm	11.8
57	Skensved æble	8.1
58	Pigeon spejlsby	11.2
59	Borgherre	12.7
60	Mathilde æble	10.9
61	Jølbyæble	9.0
62	Gråsten Rød	12.2

63	Holstenhus	13.9
64	Nonnetit fra Als	12.3
65	Pigeon Rød Vinter	11.8
66	Pigeon fra Maribo	11.3
67	Alsisk Citronæble	12.3
68	Antonius	14.2
69	Fynsk udvalg II	11.9
70	Annas æble	10.5
71	Bedstefars æble	12.7
72	Elstar	11.8
73	Arreskov	11.1
74	Gråsten Høvdinggård	12.0
75	Barritskov madæble	12.1
76	Gråmølles æble	11.9
77	Farum æble	10.7
78	Ildrød Pigeon	12.7
79	Dronning Louise	13.5
80	Risskov Rambour	10.8
81	Tagesminde æble	10.5
82	Apple 207 Knuthenborg	11.2
83	Mormors æble	13.1
84	Mølleskov	12.3
85	Herfølge voksæble	10.7
86	Lise Legind	11.0

Table S2. List of 65 compounds identified by GC-MS analysis.

Aroma compound	Mean[a]	%CV	Min	Max	Range
Alcohols					
2-methyl-1-butanol	0.0340	93	0.0000	0.1397	0.1397
3-methyl-1-butanol	0.0822	84	0.0066	0.3414	0.3347
2-methyl-1-propanol	0.2183	58	0.0317	0.6942	0.6626
2-butanol	0.0031	85	0.0005	0.0162	0.0157
2-ethyl-1-hexanol	0.0036	198	0.0005	0.0597	0.0592
2-heptanol	0.0004	154	0.0000	0.0027	0.0027
2-hexen-1-ol	0.0799	100	0.0029	0.3339	0.3310
2-pentanol	0.0007	206	0.0000	0.0091	0.0091
3-methyl-3-butenol	0.0007	123	0.0000	0.0046	0.0046
3-octanol	0.0156	119	0.0000	0.0876	0.0876
cis-3-hexenol	0.0113	122	0.0003	0.0784	0.0781
6-Methyl-5-hepten-2-ol	0.0117	124	0.0000	0.0907	0.0907
heptanol	0.0094	111	0.0000	0.0774	0.0774
hexanol	0.0017	101	0.0000	0.0075	0.0075
linalool	0.0011	280	0.0000	0.0284	0.0284
octanol	0.0080	258	0.0004	0.1860	0.1856
propanol	0.0126	101	0.0006	0.0740	0.0734
butanol	1.5807	49	0.1058	3.7960	3.6902
Esters					
2-methylbutyl acetate	0.0066	187	0.0000	0.0541	0.0541
2-methylpropyl acetate	0.0869	193	0.0000	0.9989	0.9989
hexyl acetate	0.2970	173	0.0039	1.9643	1.9604
pentyl acetate	0.0710	197	0.0000	0.5813	0.5813
propyl acetate	0.0266	210	0.0000	0.4178	0.4178
ethyl acetate	0.2693	174	0.0015	1.9708	1.9692
butyl acetate	1.1010	168	0.0066	7.2872	7.2805
methyl acetate	0.0040	240	0.0000	0.0554	0.0554
propyl 2-methylbutanoate	0.0079	231	0.0000	0.1185	0.1185
2-methylpropylbutanoate	0.0036	113	0.0000	0.0155	0.0155
butyl butanoate	0.1867	110	0.0006	1.0776	1.0770
hexyl butanoate	0.1175	120	0.0000	0.6402	0.6402
methyl 2-methylbutanoate	0.0018	216	0.0000	0.0246	0.0246
butyl 2-methyl butanoate	0.0013	118	0.0000	0.0090	0.0090
propyl butanoate	0.0945	142	0.0000	0.6770	0.6770
butyl propanoate	0.0002	198	0.0000	0.0032	0.0032
ethyl 2-methylpropanoate	0.0013	259	0.0000	0.0169	0.0169
methyl propanoate	0.0019	268	0.0000	0.0431	0.0431
ethyl 2-methylbutyrate	0.0754	219	0.0000	0.8310	0.8310
ethyl butyrate	0.4822	172	0.0000	3.9019	3.9019
hexyl-2-methyl butyrate	0.0336	125	0.0000	0.2583	0.2583
methyl butyrate	0.0721	160	0.0000	0.5141	0.5141
methyl hexanoate	0.0005	259	0.0000	0.0088	0.0088
ethyl hexanoate	0.0108	377	0.0000	0.2687	0.2687
butyl hexanoate	0.0168	151	0.0000	0.1388	0.1388
ethyl pentanoate	0.0004	303	0.0000	0.0089	0.0089
ethyl propanoate	0.0135	231	0.0000	0.1881	0.1881
ethyl-2-butenoate	0.0022	322	0.0000	0.0529	0.0529
ethyl octanoate	0.0001	213	0.0000	0.0017	0.0017
Aldehydes					
butanal	0.1362	116	0.0064	0.7854	0.7790
decanal	0.0013	155	0.0000	0.0176	0.0176
furfural	0.0014	171	0.0000	0.0124	0.0124
heptanal	0.0007	91	0.0000	0.0052	0.0052
hexanal	0.6609	69	0.0394	2.2950	2.2555
nonanal	0.0048	174	0.0013	0.0772	0.0758
octanal	0.0021	146	0.0000	0.0287	0.0287
pentanal	0.0035	152	0.0000	0.0368	0.0368
t-2-hexenal	0.0990	60	0.0000	0.2931	0.2931
2-methylbutanal	0.0012	98	0.0000	0.0071	0.0071
Ketons					
3-octanone	0.0012	116	0.0000	0.0064	0.0064
2-butanone	0.0035	85	0.0000	0.0214	0.0214

2-heptanone	0.0003	178	0.0000	0.0030	0.0030
2-nonanone	0.0001	264	0.0000	0.0012	0.0012
2-propanone	0.0094	61	0.0020	0.0426	0.0406
6-methyl-5-hepten-2-one	0.0042	82	0.0000	0.0180	0.0180
Terpenes					
α-pinene	0.0021	457	0.0000	0.0801	0.0801
farnesene	0.0277	194	0.0000	0.3968	0.3968

[a]Expressed in arbitrary units a.u.=peak area/internal standard peak area

Table S3. Distinct odors and flavors of the 86 juices and numerical evaluation of their sensory attributes.

#	Apple cultivar	Distinct odour	Distinct flavour	Overall odour	Brown colour	Overall flavour	Apple flavour	Sweet taste	Sour taste
1	Louisendal		subacid fruit/watery	9.8	7.1	7.1	6.8	6.1	6.7
2	Skovfoged			6.8	6.4	8.0	8.6	7.6	7.8
3	Ferskenrødt sommeræble	apricot	peach	9.3	6.7	9.2	8.8	9.4	7.1
4	Ejby æble		watery	8.3	7.3	7.3	7.4	6.3	5.2
5	Augustæble	chemical	watery	7.9	8.6	6.7	7.2	7.4	5.6
6	Uggerløse æble	berries	rhubarb/subacid fruit	6.4	0.7	8.0	7.2	7.2	9.1
7	Herschendsgave	berries/complex	complex/fresh apple/peach	7.9	8.3	9.5	9.5	9.8	6.6
8	Gadeskovæblet		sour	7.0	3.7	7.3	7.7	6.7	10.1
9	Auroravej	artichoke		6.8	9.6	8.4	6.9	8.3	3.5
10	Ondrup sommeræble		citrus	6.7	0.9	9.0	8.0	9.1	8.8
11	Fåborgæble	pineapple/rhubarb/unripe black currant	sour	9.7	2.3	8.3	7.1	7.0	11.0
12	Kundbyæble		insipid/watery	8.4	10.7	7.5	6.9	7.7	4.6
13	Ingers æble			9.6	8.8	7.6	8.5	7.9	8.5
14	Ørdings æble			8.6	2.2	6.8	6.8	5.2	11.6
15	Ulderup æble			8.0	6.2	8.2	8.0	8.0	7.5
16	Thyregod kalvil		sour	5.7	7.9	8.3	8.1	7.5	8.6
17	Fuhræble	berries/mandarin/peach	mandarin/peach	7.9	4.2	9.0	7.4	8.2	8.6
18	Guldspir		bitter	7.7	4.0	7.8	8.0	7.2	9.2
19	Gravenfin	peach/pear	subacid fruit	9.2	8.0	9.1	8.7	9.0	8.1
20	Sofie æble		subacid fruit/bitter	7.4	6.1	8.0	8.1	7.1	9.3
21	Nina's æble		subacid fruit	8.3	5.5	9.7	9.3	8.9	8.3
22	Søde æbler fra Aalsrode	complex		9.8	10.1	8.0	6.7	7.4	2.4
23	Vallekilde Sommeræble		fresh apple/watery/ bitter	7.2	4.1	7.8	8.3	8.2	6.3
24	Rosenholm	fresh green		7.9	4.8	9.3	8.9	9.3	7.8
25	Dynnegårdsæble	over ripe	full-bodied	8.3	7.3	9.6	8.8	8.9	7.1
26	Nonnetit Bastard			8.5	4.6	8.9	8.4	7.8	8.5
27	Miang æble		watery	7.6	9.0	9.4	8.8	9.1	7.1
28	Gråsten Gul			7.8	6.7	8.4	7.7	8.7	7.3
29	Pigeon Stribet			7.7	7.0	8.9	9.1	9.2	7.3
30	Butteræble	grapefruit	complex fruit/grape fruit	9.5	5.8	9.6	6.9	9.3	6.5
31	Vejløæble			8.0	6.5	7.7	7.6	7.4	7.5
32	Nybøllegård		watery	8.1	5.8	8.4	8.7	7.9	6.2
33	Fejø æble		full-bodied	7.3	5.7	10.2	9.7	9.6	8.1
34	Hindbæræble		subacid fruit/ bitter	9.0	7.2	8.3	8.5	8.2	7.9
35	Pilehavesæble		citrus/sour	6.4	0.9	8.8	7.8	8.3	10.0
36	Langt rødt Hinbæræble		bitter	8.6	7.1	9.2	8.9	9.4	6.3
37	Mosede æbler			7.7	7.0	6.9	7.0	7.2	5.8
38	Ingrid Marie			8.6	3.9	8.0	8.2	8.0	7.5
39	Maglemer rød			5.1	4.2	7.2	6.7	7.4	6.3
40	Fynsk udvalg V		watery	9.3	5.7	6.8	7.0	7.0	7.5
41	Lundbytorp æble		full-bodied	8.5	10	10.3	10.1	10.3	5.7
42	Bodil Neergård		subacid fruit	8.2	4.8	9.1	9.2	9.5	7.7
43	Filippa Harritslev	fresh green/pineapple	complex/fresh apple	10.1	2.8	10.4	9.8	10	7.2
44	Tønnes			7.8	5.8	8.1	7.5	10.2	5.7
45	Flaskehalser	sharp		8.8	8.3	9.5	9.8	9.5	7.7
46	Flintinge		complex	9.1	3.6	10	9.4	9.2	8.4
47	Jakober			8.9	5.9	7.8	7.9	7.4	9.2
48	Æbletoftæble			6.8	4.2	8.0	7.2	8.9	6.5
49	Skenkelsø æble		sour	7.6	7.0	9.4	9.3	8.5	9.0
50	Broholm Rosenæble	complex	insipid/bitter	7.8	5.6	8.7	8.4	8.7	7.0
51	Ondrup moseæble			8.0	6.5	7.4	7.3	7.2	7.0
52	Høje Taastrup æble			8.0	6.4	7.5	7.4	7.3	7.1
53	Knud Lunn	peach	subacid fruit/watery	5.6	7.0	8.4	7.9	7.2	8.7
54	Niels Juul			9.8	7.2	9.3	9.4	9.8	5.6
55	Thyrislund			5.1	7.0	7.4	7.6	8.4	6.9
56	Broholm		fresh apple/red berries/subacid fruit/bitter	8.8	3.8	9.6	9.6	9.6	6.7
57	Skensved æble		watery	7.6	9.1	5.5	6.6	6.0	4.8
58	Pigeon spejlsby		bitter	8.5	8.4	8.6	8.0	9.1	6.5
59	Borgherre	weak	citrus/fresh apple/pear	6.4	1.8	10.2	8.6	9.5	7.6
60	Mathilde æble	peach		9.5	7.3	8.9	8.7	9.2	6.7
61	Jølbyæble			8.0	6.5	7.7	7.6	7.3	7.4
62	Gråsten Rød			8.5	6.1	8.8	7.5	8.4	5.6
63	Holstenhus		bitter	5.0	4.8	8.5	8.7	10.3	8.0
64	Nonnetit fra Als	chemical/pear	chemical/fresh apple	8.6	4.3	9.6	8.5	9.0	6.8
65	Pigeon Rød Vinter	strawberry	strawberry/bitter	6.2	8.8	9.1	8.9	8.4	8.1
66	Pigeon fra Maribo			9.3	8.2	9.1	9.1	8.8	8.7
67	Alsisk Citronæble	pineapple/rhubarb/Tea	pear/pineapple/tea	8.6	6.5	8.8	8.0	8.3	6.8
68	Antonius	peach	tropical fruit	8.6	9.2	10.4	10.0	10.4	6.9
69	Fynsk udvalg II		apricot	7.4	7.4	9.4	8.9	8.3	8.0
70	Annas æble		pineapple	5.9	8.5	5.3	6.2	7.5	7.7
71	Bedstefars æble	fresh green	fresh apple	9.3	6.3	9.6	9.9	9.1	7.7
72	Elstar			8.1	5.9	8.5	8.2	8.4	7.0
73	Arreskov		watery	8.7	5.5	7.2	7.8	7.9	5.4

74	Gråsten Høvdinggård			7.4	3.6	4.9	6.6	6.9	6.2
75	Barritskov madæble	weak	complex/pineapple	8.7	1.2	9.1	8.1	9.0	7.8
76	Gråmølles æble		red berries/bitter	7.1	4.9	9.3	8.4	7.7	7.5
77	Farum æble	pear	pear/watery/bitter	7.4	6.6	7.8	7.0	6.7	9.4
78	Ildrød Pigeon	complex	bitter/subacid fruit/bitter	8.1	7.5	7.8	7.9	7.7	7.9
79	Dronning Louise	pear	full-bodied	10.1	9.5	10.2	10.0	10.0	6.3
80	Risskov Rambour		watery	8.3	7.9	8.4	9.0	7.7	8.9
81	Tagesminde æble		sour/watery	6.3	2.8	7.7	7.6	7.2	9.3
82	Apple 207 Knuthenborg	pear		7.4	6.2	8.6	8.8	8.9	6.9
83	Mormors æble	apricot/sweetish fruit	apricot/complex/peach	7.6	6.3	10.0	9.7	9.8	5.6
84	Mølleskov	over ripe	watery	8.7	4.7	9.8	8.6	9.2	7.6
85	Herfølge voksæble	fresh green	citrus	6.3	2.9	8.5	7.6	6.1	9.6
86	Lise Legind		peach/strawberry/watery	8.7	6.3	8.3	8.5	8.4	6.4

Table S4. Average concentrations of the compounds and parameters identified from Bruker SGF profiling.

	units	LOQ[a]	mean	SD[b]	%CV[c]	min	max	range	A.I.J.N. (Apple)	
									min	max
Sugars										
Sucrose	g L^{-1}	0.2	31.8	11.9	37.5	7.5	66.8	59.3	5.0	30.0
Glucose	g L^{-1}	0.5	12.3	3.8	31	5.6	21.1	15.5	15.0	35.0
Fructose	g L^{-1}	0.5	51.3	7.9	15	28.9	67.9	38.9	45.0	85.0
Xylose	mg L^{-1}	300	421.7	162.8	38.6	N.D.[c]	863	-	-	-
Acids										
Malic	g L^{-1}	0.5	9.7	2.5	25.2	2.5	18.9	16.4	3.0	-
Cholorogenic	mg L^{-1}	20	37.1	60.2	162	N.D.	377	-	-	-
Succinic	mg L^{-1}	10	6.9	10.3	147.7	N.D.	41	-	-	-
Citramalic	mg L^{-1}	10	13.4	15.4	115	N.D.	71	-	-	-
Quinic	mg L^{-1}	50	573.1	349.4	61	151.0	2520	2369	-	-
Quality indicators										
Acetaldehyde (*n=1*) *	mg L^{-1}	5	-	-	-	-.	8	-	-	-
Alanine (*n=69*) *	mg L^{-1}	5	19.7	15.8	80	5.0	73.0	68	1	50
Ethanol (*n*=34) *	mg L^{-1}	10	88.0	92.0	105	10	371	361	-	3000
Galacturonic acid (*n*=2) *	mg L^{-1}	100	136.5	12.0	8.8	128	145	17	-	-
Methanol (*n=21*) *	mg L^{-1}	10	16.9	8.9	52.6	10.0	47.0	37	-	-
Lactic acid	mg L^{-1}	10	-	-	-	-	-	-	-	500
Acetoine	mg L^{-1}	10	-	-	-	-	-	-	-	-
Arbutin	mg L^{-1}	10	-	-	-	-	-	-	-	-
Benzaldehyde	mg L^{-1}	5	-	-	-	-	-	-	-	-
Citric acid	mg L^{-1}	0.5	-	-	-	-	-	-	-	0.1
Benzoic acid	mg L^{-1}	10	-	-	-	-	-	-	-	-
Formic acid	mg L^{-1}	5	-	-	-	-	-	-	-	-
Fumaric acid	mg L^{-1}	5	-	-	-	-	-	-	-	5
Proline	mg L^{-1}	50	-	-	-	-	-	-	-	-
Pyruvic acid	mg L^{-1}	10	-	-	-	-	-	-	-	-
Sorbic acid	mg L^{-1}	10	-	-	-	-	-	-	-	-
5-hydroxymethylfurfural	mg L^{-1}	5	-	-	-	-	-	-	-	20
Ratios										
Malic/quinic			21.3	11.0	51.8	5.1	68.3	63.2	-	-
Glucose/Fructose			0.24	0.065	27.3	0.10	0.42	0.32	0.30	0.50
Total sugar	g L^{-1}	2	95.4	12.0	12.5	62.5	126.1	63.6	-	-

[a]LOQ = Limit of Quantification.

[b]SD = Standard Deviation.

[c]%CV = percent Coefficient of Variation calculated as (SD/mean) *100

[d]N.D. = Not Detectable.

* The statistical parameters have been calculated on the *n* indicated in brackets. The remaining samples showed concentrations below the LOQ.

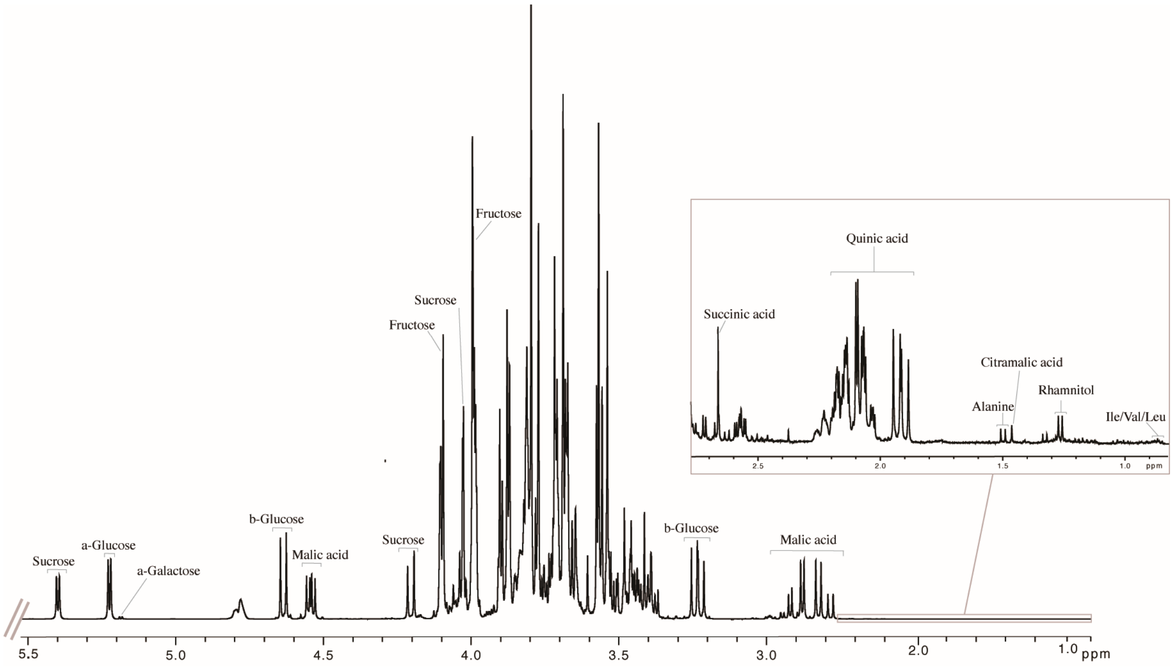

Figure S1. Signal assignment of a representative ^{1}H-NMR spectrum of apple juice (0 – 5.5 ppm).

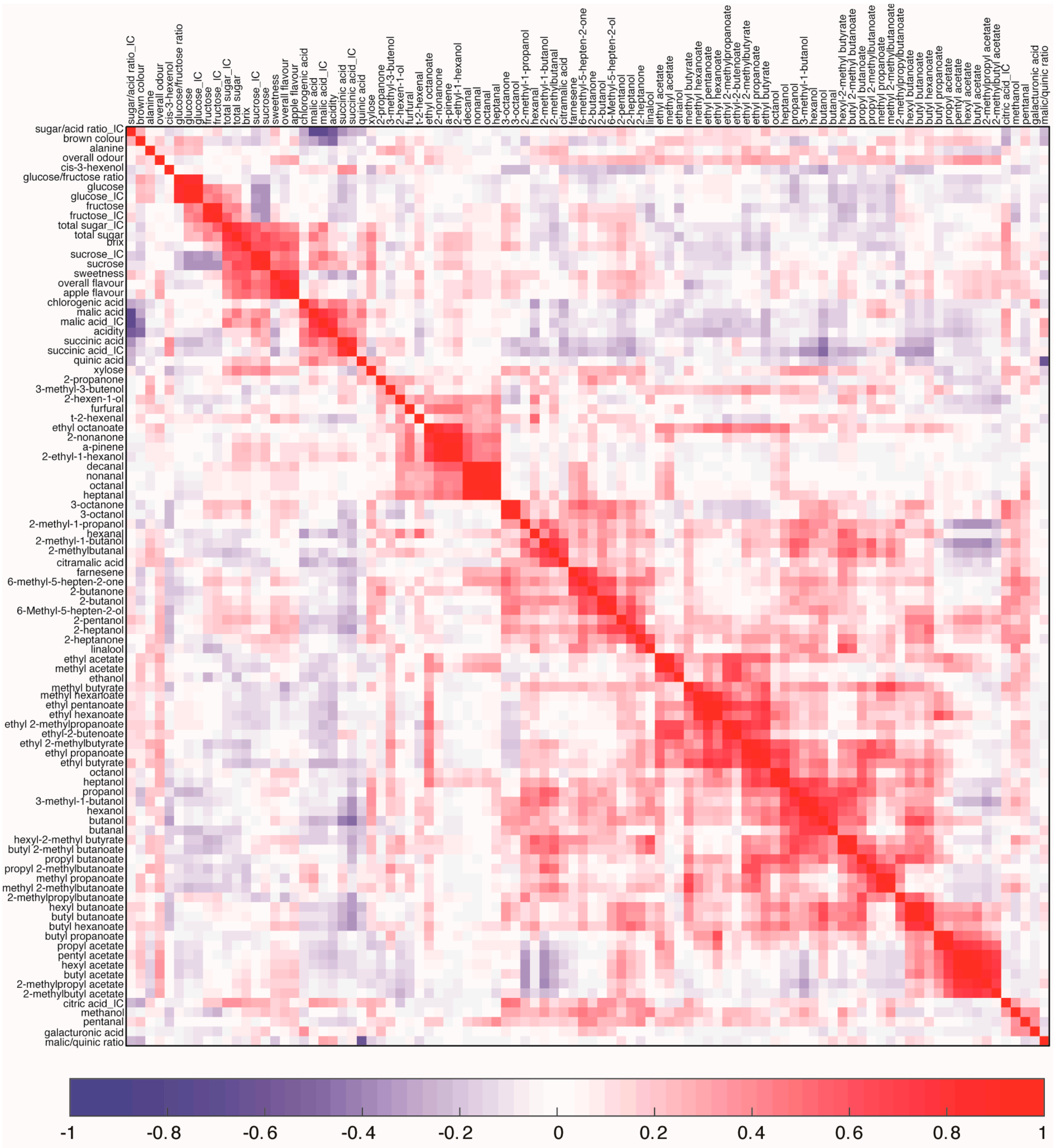

Figure S2. Correlation map generated from the 86 x 96 matrix. The variables are grouped by similarity.

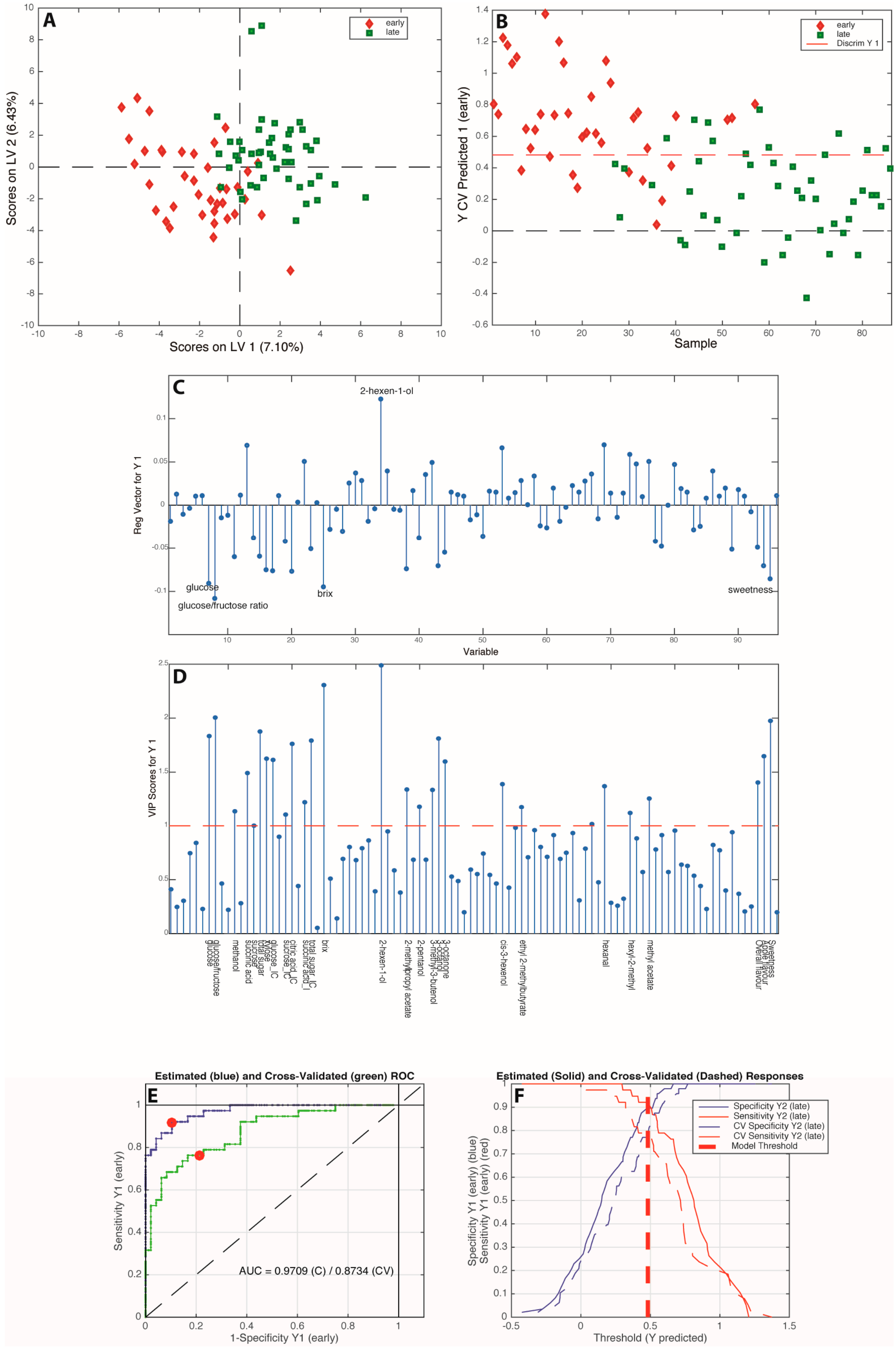

Figure S3. (A) LV1/LV2 scores plot of the PLS-DA model developed to discriminate early-season juices (class 1) from late (class 2) harvest cultivars. Most discriminative markers are shown in the regression vector plot (C) and VIP score plot (D). Area Under the Curve (AUC) of Receiver Operating Characteristic (ROC) (E) and sensitivity and specificity (F) plot of the PLS-DA model.
